# Culture media influences *Candida parapsilosis* growth, susceptibility, and virulence

**DOI:** 10.3389/fcimb.2023.1323619

**Published:** 2023-12-13

**Authors:** Betsy V. Arévalo-Jaimes, Joana Admella, Núria Blanco-Cabra, Eduard Torrents

**Affiliations:** ^1^ Bacterial Infections and Antimicrobial Therapies Group (BIAT), Institute for Bioengineering of Catalonia (IBEC), The Barcelona Institute of Science and Technology (BIST), Barcelona, Spain; ^2^ Microbiology Section, Department of Genetics, Microbiology and Statistics, Faculty of Biology, University of Barcelona, Barcelona, Spain

**Keywords:** amphotericin B, caspofungin, antifungal, pseudohyphal growth, pathogenicity, *Galleria mellonella*, biofilm, *Candida*

## Abstract

**Introduction:**

*Candida parapsilosis*, a pathogenic yeast associated with systemic infections, exhibits metabolic adaptability in response to nutrient availability.

**Methods:**

We investigated the impact of RPMI glucose supplemented (RPMId), TSB, BHI and YPD media on *C. parapsilosis* growth, morphology, susceptibility (caspofungin and amphotericin B), and *in vivo* virulence (*Galleria mellonella*) in planktonic and biofilm states.

**Results:**

High-glucose media favors growth but hinders metabolic activity and filamentation. Media promoting carbohydrate production reduces biofilm susceptibility. Virulence differences between planktonic cells and biofilm suspensions from the same media shows that biofilm-related factors influence infection outcome depending on nutrient availability. Pseudohyphal growth occurred in biofilms under low oxygen and shear stress, but its presence is not exclusively correlated with virulence.

**Discussion:**

This study provides valuable insights into the intricate interplay between nutrient availability and *C. parapsilosis* pathogenicity. It emphasizes the importance of considering pathogen behavior in diverse conditions when designing research protocols and therapeutic strategies.

## Introduction

*Candida parapsilosis* is an important pathogen in systemic infections, particularly prevalent in Latin America, parts of Asia, and Southern Europe. It is known for its ability to colonize medical devices and thrive in high-glucose environments, posing a threat to vulnerable patient groups including neonates, the elderly, and surgically treated individuals. Infections are often acquired from hands of healthcare workers or through prolonged use of total parenteral nutrition, central venous catheters, and other medically implanted devices (Tóth et al., 2019; Rupert and Rusche, 2022).

The formation of *C. parapsilosis* biofilms plays a critical role in patient outcomes, as these aggregates of blastospores and/or pseudohyphae, surrounded by an extracellular matrix (ECM) rich in carbohydrates, have been linked to increased morbidity, mortality, and resistance to conventional therapies (Tóth et al., 2019; Konečná et al., 2021). However, the role of pseudohyphal growth in *C. parapsilosis* pathogenicity remains relatively unexplored (Silva et al., 2009; Tóth et al., 2018; Banerjee et al., 2019). In contrast, the pathogenicity of *C. albicans* hyphae has been associated with the colonization and invasion process of epithelial barriers, major immune responses induction, and host cell damage via toxin secretion, among others (Silva et al., 2011; Lackey et al., 2013; Tóth et al., 2019).

*In vitro* biofilm studies of non-*albicans Candida* species should consider various culture variables, such as the inoculum concentration, temperature, levels of O_2_ and CO_2,_ incubation period, growth media, and feeding conditions, as yeasts can adapt their metabolism based on nutrient availability, leading to changes in virulence factor expression, and stress-inducing environmental stimuli can influence morphology transition (Tan et al., 2016; Tóth et al., 2019; Konečná et al., 2021).

This study aims to investigate whether the effect of culture media on *C. parapsilosis* pathogenicity is attributed to metabolic or morphological adaptations, particularly pseudohyphal growth, induced by nutrient composition. We comprehensively assess the impact of nutrient availability on key biological traits of *C. parapsilosis*, utilizing four different culture media to evaluate growth, morphology, antifungal susceptibility, and *in vivo* virulence (using the *G. mellonella* model) of both planktonic and biofilm states. Our findings underscore the importance of establishing standardized culture conditions that mimic the natural infection environment to avoid cofounding alterations in biofilm properties. Overall, our study provides valuable insights into the intricate relationship between nutrient availability and *C. parapsilosis* pathogenicity.

## Materials and methods

### Bacterial strains and growth conditions

The fungaemia clinical isolate *Candida parapsilosis* 11103595 (Marcos-Zambrano et al., 2014) was stored at -80°C, and a loophole was recovered weekly to avoid genetic and epigenetic changes resulting from multiple passages. Incubation at 30°C for 24 h was performed on Yeast Peptone Dextrose (YPD) Agar: 1% Yeast Extract (Gibco, USA), 2% Meat Peptone (Scharlau, Spain), 2% D-glucose (Fisher Scientific S.L., USA), and 2% Bacteriological Agar (Scharlau, Spain).

Four different culture media commonly used in yeast biofilm studies (Lackey et al., 2013; Pereira et al., 2015; Tan et al., 2016; Tan et al., 2017; Leonhard et al., 2018; Gómez-Molero et al., 2021) were selected and ranked in terms of nutritional content, from highest to lowest: 1) YPD, a complex medium that supports yeast growth without selective conditions (Tan et al., 2016). It contains yeast extract and peptone that stimulate fungal replication. 2) Brain Heart Infusion (BHI) (Scharlau, Spain), a complex medium nutritionally rich but with lower glucose content than YPD. 3) Tryptic Soy Broth (TSB) (Scharlau, Spain), a complex medium with high peptone content and glucose levels similar to BHI. 4) RPMI-1640 with L-glutamine without sodium bicarbonate (Sigma-Aldrich, USA) supplemented with D-glucose at 0.2%, buffered at pH 7.0 and filter-sterilized, referred to as RPMId. A synthetic defined medium less nutritionally rich than the previous ones, but with the required amino acids and glucose content for yeast growth (Tan et al., 2016; Tan et al., 2017; Konečná et al., 2021).

### Biofilm formation on silicone coupons

To obtain the inoculum for biofilm formation, yeast suspensions in each medium were prepared from YPD Overnight cultures (ON) (~16 h) at 200 rpm and 30°C. First, cells were recovered by a centrifugation at 4000, rpm for 5 min. Then, two washes with Phosphate Buffer Saline 1X (PBS) (Fisher Scientific S.L., USA) were performed. Finally, dilutions to an optical density of λ = 550 nm (OD_550_) of 0.15 were made in each medium supplemented with 10% Fetal Bovine Serum (FBS) (Thermofisher, USA).

Silicone squares (area 1 cm^2^, thickness 1.5 mm ± 0.3mm; Merefsa, Spain) were sterilized by autoclave and pre-treated with FBS at 37°C ON. A wash with PBS was performed before placing them in 24-wells cell culture plates (SPL Life Sciences, South Korea) filled with 600 µL/well of the previously prepared yeast suspensions. Biofilms were formed on a fed-batch condition by placing the silicone squares on a flat position at the bottom of the well. An exception was made at the morphological characterization experiment, where silicon squares were placed in a diagonal position inclined against the wall of the well, creating an Air Liquid Interphase (ALI) zone and a Bottom zone on the same surface.

Then, an adhesion step of 90 min was performed at 37°C and 60 rpm. Silicone squares were dipped in PBS to remove unattached cells and carefully passed to a new 24-well plate with the respective fresh media. Finally, incubation in the same conditions was carried out during 42-48 h with an intermediate step of media renewal at 24 h to create the fed-batch condition.

In addition, flow biofilms were formed using an in-house flow chamber for silicone disks (diameter 1.1 cm, thickness 1.5 mm ± 0.3mm; Merefsa, Spain) to explore morphological changes associated with shear stress and continuous media replacement. The flow chamber was connected as previously described for a continuous-flow cell experiment (Cendra et al., 2019) with a constant flow rate of 70 mL/min of each medium tested for 24 h at 37 °C ± 3 °C. Silicone disks were pre-treated with FBS by injection into the system overnight, and a 2 h-adhesion step was performed after yeast suspension inoculation.

### Morphological microscopic evaluation

Morphological characterization of planktonic cells and biofilms grown in the four media was performed. To this end, ON cultures were grown in the four culture media at 30°C, the standard temperature for yeast growth in laboratory settings, and at 37°C, the natural body temperature. Moreover, the effect of FBS 10% supplementation in planktonic cultures incubated at 37°C was also tested. Cells were recovered and washed twice with PBS (4000 rpm x 5 min). PBS-resuspended yeasts were imaged under a 100x magnification with a Nikon inverted fluorescent microscope ECLIPSE Ti–S/L100 (Nikon, Japan) coupled with a DS-Qi2 Nikon camera (Nikon, Japan) in Bright Field.

On the other hand, *C. parapsilosis* biofilms formed on the fed-batch condition (ALI and Bottom zone) and the continuous flow system were visualized via chitin staining with 10 µM Calcofluor white (CW) (Biotium, USA) under a Zeiss LSM 800 confocal scanning laser microscope (CSLM). Images were processed and measured using ImageJ Fiji software. Briefly, the area and the Length-to-Width Ratio (LWR, denoted as aspect ratio in ImageJ) were calculated from randomly chosen cells (n=30) from planktonic and biofilm conditions. Pseudohyphae were defined as forming chains of cells with box-shaped ends and LWR greater than three, and blastospores as cells oval-shaped with a lower LWR (Rupert and Rusche, 2022).

### Growth, metabolic activity, and susceptibility assays

The growth of planktonic cells at 37°C and 150 rpm in the different media was recorded on 96-well polystyrene plates with a flat bottom (Corning, USA) for 15 h in a SPARK Multimode Microplate Reader (Tecan, Switzerland). Absorbance readings at 550 nm were taken every 15 min.

The minimum inhibitory concentration of 50% (MIC_50_) for caspofungin diacetate (CAS) (Merck Life Science, Spain) and amphotericin B (AmB) (Gibco, USA) is defined as the concentration of the antifungal that inhibited growth by 50%, and it was previously determined for the employed *C. parapsilosis* isolate in our laboratory as 2 mg/mL and 0.125 mg/mL, respectively. To evaluate susceptibility variations due to media composition, planktonic cultures were grown on 96-well polystyrene plates with a flat bottom, with each media supplemented with 4 and 2 mg/mL of CAS and 0.5, 0.25 and 0.125 mg/mL of AmB. Controls were treated with culture media without antifungals. Absorbance readings at 550 nm were taken every 15 min for 10 h on a SPARK Multimode Microplate Reader and MIC_50_ of each media was determined.

As the metabolic activity of *C. parapsilosis* biofilms could be reduced by echinocandins, but resistance against azoles and standard formulations of AmB has been reported (Tóth et al., 2019), we treated 42 h mature biofilms formed on each medium with concentrations of CAS MIC_50_ x 5 (10 mg/mL) and AmB MIC_50_ x 10 (1.25 mg/mL). Control squares were treated with culture media without antifungals. 6 h after treatment, Crystal Violet (CV) (Merck Life Science, Spain) assay or PrestoBlue Cell Viability assay (Thermo Fisher Scientific, USA) were performed to quantify biomass and metabolic activity, respectively. Briefly, biofilm biomass quantification was assessed by CV 0.1% (w/v) staining for 5 min followed by a distaining step with acetic acid glacial (Scharlau, Spain) at 33% (v/v). Typical washing and fixation steps were omitted due to easy biofilm detachment from the silicone surface. OD_570_ was measured in a Microplate spectrophotometer Benchmark Plus (Bio-Rad, USA). Moreover, a CV assay was performed on biofilms after the adhesion step to evaluate differences in *C. parapsilosis* adhesion ability according to growth media (t= 0 h).

To assess biofilm metabolic activity, silicone squares were placed in tubes containing 1 mL of PBS, and biofilms were resuspended by vortex for 1 min thrice, followed by 5 min submersion in an Ultrasonic cleaner Branson2000, (Branson Ultrasonics, Netherlands). A solution 1:10 of the resazurin-based reagent PrestoBlue in RPMId media was added to each biofilm suspension. After 30 min at 37°C in the dark, fluorescence (λ_Exc_= 535 nm and l_em_=615 nm) was measured in a SPARK Multimode microplate reader. In addition, to corroborate the metabolic activity evaluation, biofilms were stained with FUN-1 (Thermo Fisher Scientific, USA), a green intracellular dye that converts to orange-red fluorescent cylindrical intravacuolar structures (CIVS) when cells with intact membranes have metabolic activity (Millard et al., 1997). FUN-1 at 20 mM and CW at 10 mM were added, and biofilms were incubated for 30 min at 30°C and 60 rpm after 15 h of treatment. CLSM images at 63x were processed by ImageJ software.

Finally, biofilm carbohydrate content from non-treated samples was broadly estimated from 48 h biofilms formed on each media by staining α-mannopyranosyl and α-glucopyranosyl residues from polysaccharides present in the extracellular matrix (ECM) and the fungal cell wall with 25 mg/mL Concanavalin A-Alexa Fluor 647 (ConA-A647) (Invitrogen, USA) (Shopova et al., 2013) plus CW 10 mM. CLSM images at 40X were processed by Fiji-ImageJ software. Quantification was performed with COMSTAT2 plugin from Image J software.

### *Galleria mellonella* maintenance and *in vivo* virulence testing

*G. mellonella* larvae were fed with an artificial diet (15% corn flour, 15% wheat flour, 15% infant cereal, 11% powdered milk, 6% brewer’s yeast, 25% honey, and 13% glycerol) and reared at 34 °C in darkness (Moya-Andérico et al., 2021).

Yeast suspensions from ON planktonic cultures in each medium were diluted in PBS, and 10 mL of three different concentrations (1x10^7^, 5x10^7^ and 1x10^8^ CFUs/mL) were injected into the hemocoel of eight 200 mg larvae per group, through the second left proleg using a 26-gauge microsyringe (Hamilton, Reno, NV, USA). CFUs were counted by serial plating on YPD agar and incubated for 24 h at 30°C. A control group was injected with 10 mL of PBS. All larvae were incubated at 37 °C, and mortality was monitored during 16-48 h post-injection with observations done at 16, 20, 24, 38, 42, 46 and 48 h. The same procedure was performed to determine the *in-vivo* virulence of biofilm suspensions from 24 h biofilms grown on each media in the fed-batch system. Briefly, silicon squares were placed in tubes containing 1 mL of PBS. Biofilms were resuspended by vortex during 1 min thrice, followed by 5 min on the Ultrasonic cleaner Branson2000. Biofilm suspensions were subjected to an additional vortex immediately before each larvae inoculation.

### Statistical analysis

**Figure 1:**
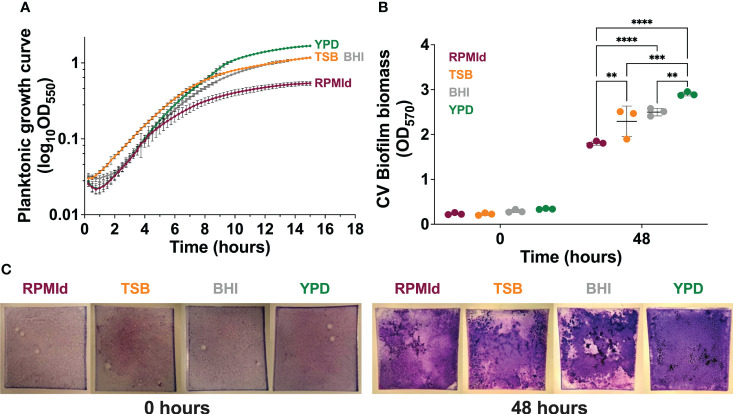
Influence of culture media on *C. parapsilosis* growth. **(A)** Planktonic growth curves on different culture media by absorbance measurements taken every 15 min for 15 h. The absorbance of each respective media was subtracted from each curve, and mean lines and standard deviation bars from 3 different experiments (4 wells per media) are presented. **(B)** Biofilm biomass quantification on each media by Crystal violet (CV) assay after the adhesion step (0 h) and biofilm maturation process (48 h). The plotted values represent 3 different biological experiments, with each value corresponding to the mean derived from 3-6 technical replicates. Error bars display mean and standard deviation across the biological replicates. Data was compared by a two-way ANOVA analysis with a Tukey’s multiple tests (∗∗*p*-value <0.01; ∗∗∗*p*-value <0.001; ∗∗∗∗*p*-value <0.0001). **(C)** Representative macroscopic images of silicone squares dyed with CV after the adhesion step (0 h) and biofilm maturation process (48 h) on each media.

**Figure 2:**
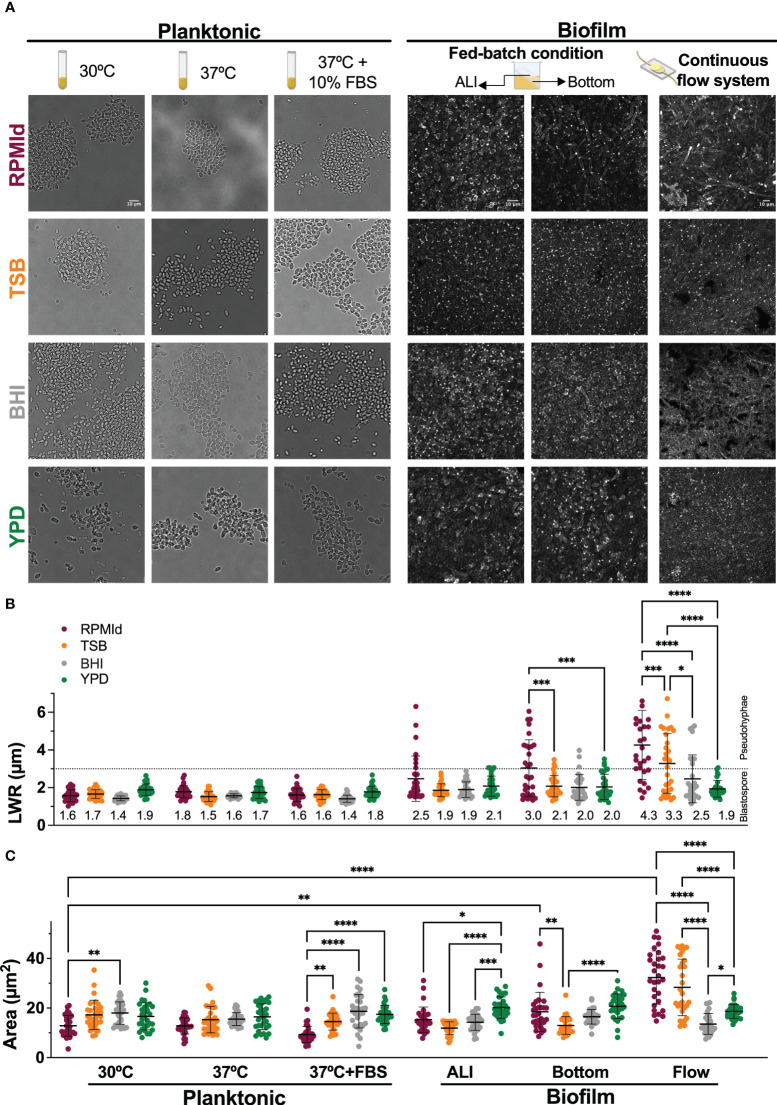
*C. parapsilosis* morphology under different media and growth conditions. **(A)** From left to right: bright-field microscopy images of overnight cultures grown in four different media at 30°C and 37°C without and with 10% of Fetal Bovine serum (FBS) supplementation; 48h biofilms grown on silicone squares placed in an inclined position in 24-wells plates to obtain an Air Liquid Interphase (ALI) and a Bottom zone; Biofilms formed on silicone coupons under continuous flow inside a custom-made device at 70 µL/min for 24h. Confocal images of biofilms were taken using Calcofluor White dye and processed with Image-J to create Z-stacks of top layers. The scale bar of 10 mm is the same for each growth condition. **(B)** Length-to-wide ratio (LWR) and **(C)** area of randomly selected cells (n=30) from each media and condition measured with Image J Horizontal dashed line represents the LWR limit for morphology classification: dots below the dashed line correspond to cells considered blastospores, and dots above the line are considered pseudohyphae cells. Numbers in LWR graph correspond to mean value. Error bars display mean and standard deviation. Data was compared by a one-way ANOVA analysis with Šidák’s multiple comparison test (∗*p*-value <0.05; ∗∗*p*-value <0.01; ∗∗∗*p*-value <0.001; ∗∗∗∗*p*-value <0.0001).

**Figure 3:**
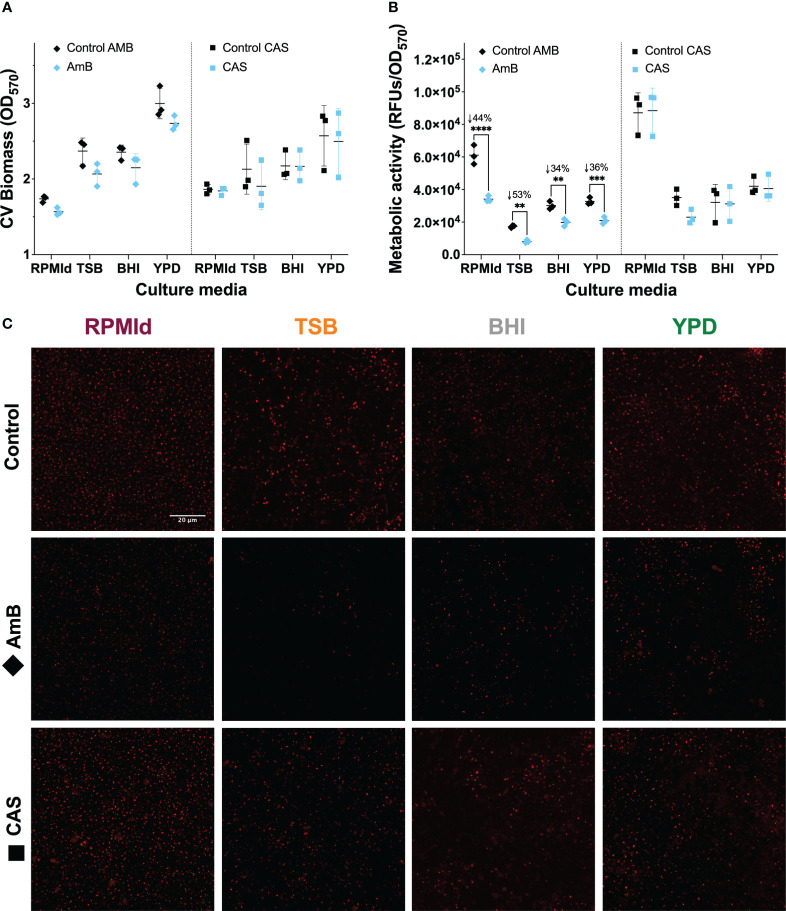
*C. parapsilosis* biofilm susceptibility to AmB and CAS at different culture media. 42 h biofilms grown in all media and treated for 6 h with 1.25 mg/mL of AmB and 10 mg/mL of CAS were analyzed by **(A)** Biomass quantification with Crystal Violet (CV) assay, and metabolic activity evaluation by **(B)** PrestoBlue assay, and **(C)** FUN-1 vitality staining. Biofilm experiments were conducted in triplicate. The plotted values represent 3 different biological experiments, with each value corresponding to the mean derived from 3-6 technical replicates. Error bars display mean and standard deviation. Asterisks indicate statistically significant differences versus control in an Ordinary two-way ANOVA with Šidák’s multiple comparison test (∗∗*p*-value <0.01; ∗∗∗*p*-value <0.001; ∗∗∗∗*p*-value <0.0001). The percentage of reduction concerning control is indicated with the symbol ↓ on top of the asterisks. RFUs, Relative Fluorescence Units. Z-stacks of red fluorescence channel display intravacuolar structures as an indicator of metabolically active cells. All images were processed with Image-J. The scale bar (20 mm) is the same for all images.

All presented data were obtained from n = 3 independent samples and *in vivo* assays were performed using n = 8 larvae. Data were analyzed by GraphPad Prism 9.00 and are presented as mean ± standard deviation. A two-way ANOVA analysis with a Tukey’s multiple comparisons test was performed to compare biofilm biomass from each media at two different time points (**Figure 1B**). Ordinary one-way ANOVA with Šidák’s multiple comparison test was conducted to compare LWR and area of cells from cultures and biofilms grown on different media within each evaluated condition (**Figures 2B, C**). An additional comparison of the area of cells grown in RPMId in each evaluated condition was performed (**Figure 2C**). Biofilm biomass and metabolic activity from control and treated biofilm samples (**Figures 3A, B**) were compared with a two-way ANOVA analysis with Šidák’s multiple comparison. Finally, Long-rank tests were conducted between Kaplan Meyer curves to evaluate virulence differences (**Figures 5B–D**). A defined *p*-value <0.05 was considered statistically significant in all cases. Moreover, when required, a Shapiro-Wilk and Kolmogorov-Smirnov test were used to evaluate normality, and the *p*-value was automatically adjusted for multiplicity.

### Use of generative AI and AI-assisted technologies in the writing process

ChatGPT version GPT-3.5 from OpenAI was used during the preparation of this work to improve readability and language. After using this tool, the authors reviewed and edited the content as needed.

## Results

### Culture media impact *C. parapsilosis* growth and morphology

First, we assessed the impact of four commonly used media on the growth of *C. parapsilosis* planktonic cultures and biofilms formed under fed-batch conditions. YPD supported the highest planktonic growth, as evident from the absorbance curve (**Figure 1A**), followed by TSB and BHI, while RPMId showed the lowest growth. The trend was consistent in fed-batch biofilms, as seen through Crystal violet (CV) staining (**Figures 1B, C** 48 h). To ensure that media properties did not influence biofilm biomass (**Figures 1B, C** 48 h) by attachment variations, a CV assay after the adhesion step was performed (**Figures 1B, C** 0 h), showing no significant differences among media.

Next, we investigated how culture media influenced *C. parapsilosis* morphology using microscopic analysis (**Figure 2**). According to the Length-to-Wide Ratio (LWR), planktonic cultures predominantly exhibited blastopores (~1.5 µm) regardless of the culture media used (**Figures 2A, B**). RPMId-grown cells showed a significant reduction in cell size at 30°C and 37°C + FBS conditions (**Figure 2C**. Planktonic. Fuchsia dots).

More relevant morphological differences were observed in biofilms formed under three different conditions (see methods section). 1) At the Air Liquid Interface (ALI) of fed-batch biofilms, RPMId and YPD showed pseudohyphae, with a proportion of 23% and 3%, respectively (**Figures 2A, B**. Biofilm-ALI. Fuchsia and green dots). RPMId induces pseudohyphae cells with greater length (1.5-6.3 µm) (**Figure 2B**. Biofilm-ALI. Fuchsia dots), and YPD produced significatively larger cells (30%) compared to the other media (~20 µm^2^ vs ~14 µm^2^) (**Figure 2C**. Biofilm-ALI. Green dots).

2) In the Bottom zone of fed-batch biofilms, where oxygen availability was lower, pseudohyphal growth was evident in all media, but more pronounced in RPMId (**Figure 2B**. Biofilm-Bottom. Fuchsia dots), with 50% pseudohyphae compared to 3% in TSB, BHI and YPD. YPD and RPMId produced biofilms with the largest cells (20.7 µm^2^ and 18.5 µm^2^, respectively) (**Figure 2C**. Biofilm-Bottom. Green and fuchsia dots).

Finally, 3) in continuous flow biofilms with shear stress, RPMId showed a significant higher proportion of pseudohyphae (~70%), followed by TSB (~50%), while BHI had 30% and YPD had almost none (3%) (**Figures 2A, B** Biofilm-Flow). RPMId and TSB produced significant the largest cells (32.3 µm^2^ and 28.3 µm^2^, respectively) (**Figure 2C**. Biofilm-Flow. Fuchsia and orange dots).

Notably, RPMId-grown cells were longer in the continuous flow system (4.3 µm) compared to the bottom (3 µm) and ALI zone (2.5 µm) of fed-batch biofilms and planktonic cultures (~1.8 µm) (**Figure 2C**. Fuchsia dots). This difference is significative when comparing planktonic cultures at 30°C with biofilms formed at the bottom of the fed-batch systems and biofilms formed under flow condition. These results highlight the role of culture media in shaping *C. parapsilosis* growth and morphology in various conditions.

### Culture media influences *C. parapsilosis* antifungal susceptibility

Then, we assessed the impact of culture media on antifungal susceptibility of *C. parapsilosis* planktonic cultures and fed-batch biofilms. The Minimum Inhibitory Concentration 50% (MIC_50_) of planktonic cultures to caspofungin (CAS) was ≤ 2 mg/mL for all media, while for amphotericin B (AmB), it was higher in YPD (0.25 mg/mL) compared to the other media (0.125 mg/mL).

In biofilms, which can impede antifungal treatment through compound sequestering (Tóth et al., 2019), we observed distinct results compared to planktonic cultures (**Figure 3**). No effect on biomass or metabolic activity was observed in biofilms following treatment with CAS at 10 mg/mL (**Figure 3A, B**. blue squares). In contrast, AmB at 1.25 mg/mL effectively reduced the metabolic activity of biofilms from all media tested (**Figure 3A, B** blue diamonds). FUN-1 staining confirmed the metabolic activity after antifungal treatment (**Figure 3C**), showing a reduction in red fluorescent cylindrical intravacuolar structures (CIVS) in AmB-treated biofilms from all media (**Figure 3C**).

**Figure 4:**
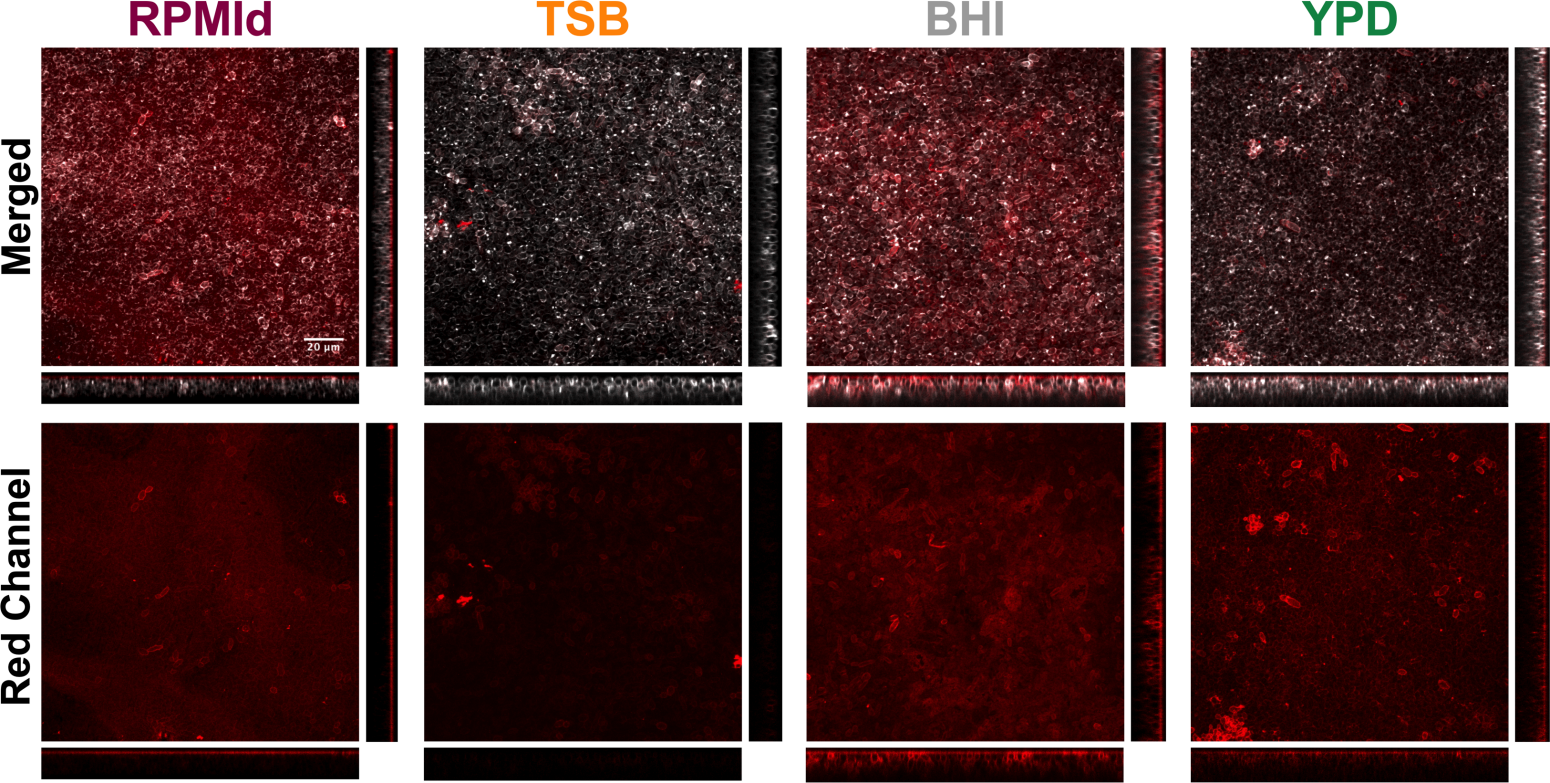
Visualization of the carbohydrate content of *C. parapsilosis* biofilms formed on different media by ConA-A647 staining. 48h biofilms grown on silicone squares with different media were stained with Calcofluor White (grey) to visualize cell walls and ConA-A647 (red) to visualize α-mannopyranosyl and α-glucopyranosyl residues from polysaccharides present in the extracellular matrix and the fungal cell wall. Images were processed with Image-J to create Z-stacks. Orthogonal views from cuts at the center of the X and Y axis are displayed. The XZ orthogonal displays deeper to superficial stacks from bottom to top, while YZ orthogonal display them from right to left. The scale bar (20 µm) is the same for all images.

To understand whether the variations in biofilm susceptibility were related to differences in the ECM, we estimated the biofilm carbohydrate content using ConA-A647 staining and COMSTAT2 quantification (**Figure 4**). The analysis revealed notable differences in carbohydrates on the cell wall and ECM, depending on the culture media used. BHI biofilms had the highest carbohydrate content (20.25 + 3.18 mm^3^/mm^2^), followed by RPMId (10.83 + 2.82 mm^3^/mm^2^), YPD (8.66 + 3.02 mm^3^/mm^2^), and TSB (2.64 + 1.93 mm^3^/mm^2^). Additionally, there was an association between biofilm carbohydrate content (**Figure 4**) and the decrease in metabolic activity after AmB treatment (**Figure 3B**). Notably, variation in the quantity of carbohydrates on the cell wall also played an important role. BHI biofilms, with a higher level of carbohydrate in both ECM and cell wall (**Figure 4**), showed a lower reduction in metabolic activity after AmB treatment (34%) (**Figure 3B**), while TSB-formed biofilms, with little carbohydrate content in the cell wall (**Figure 4**), presented the highest decline in metabolic activity (53%) (**Figure 3B**).

**Figure 5:**
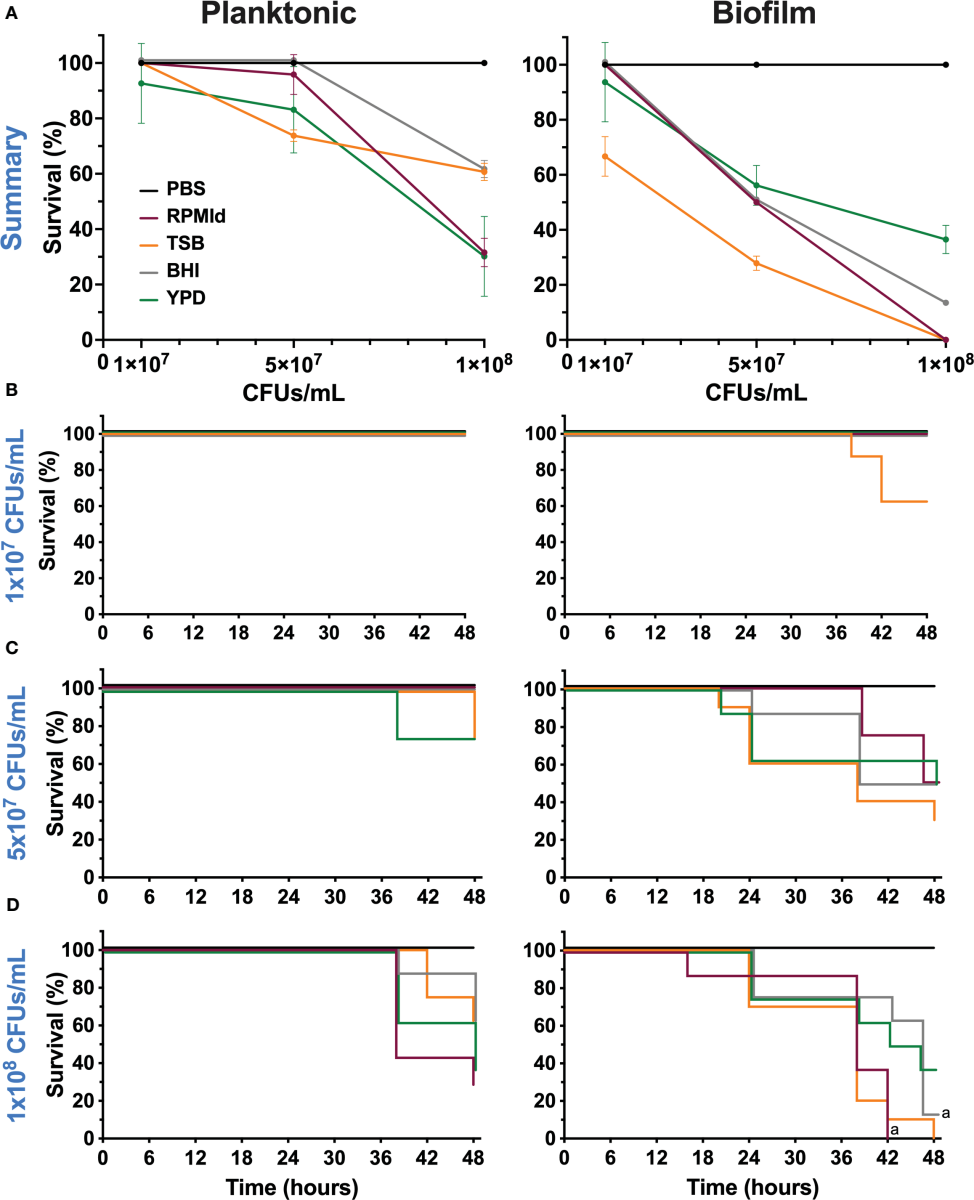
Virulence of *C. parapsilosis* cells from planktonic cultures and biofilm suspensions grown in different media on *G. mellonella* larvae. **(A)** Survival percentage of *G. mellonella* larvae at 48 h after inoculation of three different concentrations. Error bars display mean and standard deviation. Kaplan-Meier survival curves of (*G. mellonella* larvae injected with *C. parapsilosis* cells from planktonic cultures and biofilm suspensions formed on various media at concentrations of **(B)** 1x10^7^ CFUs/mL, **(C)** 5x10^7^ CFUs/mL and **(D)** 1x10^8^ CFUs/mL. Control larvae were injected with PBS. Larval mortality was monitored for 16-48 h post-injection with observations done at 16, 20, 24, 38, 42, 46 and 48 h. Significant differences among media used were evaluated by a long-rank test (a: *p*-value <0.05 between specified lines). The results presented in this figure are representative of the same experiment repeated three times with similar outcomes, each condition having 8 larvae.

### Culture media affects *C. parapsilosis* virulence

Our final aim was to evaluate variations in *C. parapsilosis* virulence according to the growth media and growth mode. To achieve this, we injected three different concentrations of planktonic cultures and PBS-resuspended fed-batch biofilms of *C. parapsilosis* grown from each medium into *G. mellonella* larvae and evaluated the survival percentage until 48 h post-infection (**Figure 5**).

In the planktonic state, we observed virulence differences depending on the culture media used when injected 5x10^7^ CFUs/mL and 1x10^8^ CFUs/mL (**Figure 5A**). There was an important decrease in survival percentage when cells from BHI, RPMId and YPD reached the highest concentration, although this decline was less pronounced in BHI. In contrast, we observed a progressive decline in the survival percentage in relation to the concentration when injecting cells from TSB.

As expected, the slope of survival curves was more pronounced when injected with biofilm suspensions than planktonic cells (**Figure 5B**). In this case, the decline in survival percentage was progressive in all media according to the inoculated concentration, but clearly, the most virulent cells came from TSB.

The Kaplan-Meier survival curves of the different concentrations of both states showed significant difference only between biofilm suspensions from RPMId and BHI at a concentration of 1x10^8^ CFUs/mL (**Figures 5B–D**).

## Discussion

The increasing incidence of *Candida*-systemic infections in the last two decades (Sharma and Chakrabarti, 2023) has boosted yeast research. However, a significant concern in yeast studies is the inconsistency in selecting culture media for growth, biofilm formation, and antifungal susceptibility testing (Konečná et al., 2021). In this study, we thoroughly evaluated the impact of four commonly used culture media in *C. parapsilosis’* main pathogenic features, both in planktonic and different biofilm growth modes. Our results clearly demonstrate specific pathogenic advantages depending on the culture media employed (**Table 1**).

**Table 1:** Summary of culture media influence on *C. parapsilosis* main characteristics.

	RPMId	TSB	BHI	YPD
Main Characteristics				
Overall nutrients	++	+++	+++	++++
Glucose content	4 g/L (0,4%)	2,5 g/L (0,25%)	2 g/L (0,2%)	20 g/L (2%)
Nitrogen content	Amino Acids »1 g/L (0.1%)	Casein and Soya peptone 20 g/L (2%)	Animal tissue peptone and Brain-Heart Infusion 27 g/L (2,7%)	Yeast extract and Meat peptone 30 g/L (3%)
Planktonic				
Growth	+	+++	+++	++++
Resistance	+	++	+	+++
Virulence	++	++	+	+++
Biofilm				
Growth	+	+++	+++	++++
Metabolic activity	++++	++	++	++
Filamentation	++++	+++	++	+
Carbohydrate content	++	+	++++	++
Resistance	+++	++	++++	++++
Virulence	+++	++++	+++	++

+ low, ++ medium, +++ high, ++++ superhigh.

The biomass quantification (**Figure 1**) showed that *C. parapsilosis* growth in planktonic and biofilm states is directly proportional to the richness of the culture medium, specifically to the high-glucose content. This finding aligns with the prevalence of *C. parapsilosis* in systemic infections of patients receiving parenteral nutrition (Nosek et al., 2009; Pereira et al., 2015; Tan et al., 2016; Herek et al., 2019; Tóth et al., 2019; Santos et al., 2020; Konečná et al., 2021). Moreover, media containing abundant nutrients and glucose inhibit pseudohyphal growth, suggesting that the transition to pseudohyphae in *C. parapsilosis* may be an evolutionarily conserved response to starvation (Lackey et al., 2013; Rupert and Rusche, 2022). Consistent with this observation, our morphological evaluation did not reveal filamentation in *C. parapsilosis* planktonic cultures (**Figure 2**. Planktonic) as we assessed only completed media. Additionally, a negligible proportion of pseudohyphae were present in biofilms formed in YPD medium, which had the highest glucose availability (**Figure 2B**. Biofilm. Green dots).

Furthermore, as reported for *C. albicans* (Thein et al., 2007; Paramonova et al., 2009), we observed that *C. parapsilosis* filamentation is promoted by low oxygen and shear conditions (**Figures 2A, B**. Biofilm-Bottom and Flow). This phenomenon is further enhanced when yeast is grown under the filament inducing medium RPMI (fuchsia dots). Moreover, in the continuous flow model, which is highly relevant considering the nature of *C. parapsilosis* infection, the degree of biofilm filamentation is inversely proportional to the richness of the media employed (**Figures 2A, B** Biofilm-Flow). This shows that the morphology of *C. parapsilosis* cells, and consequently, the composition of biofilms, is the result of multiple interacting factors. Besides, pseudohyphal growth in this *Candida* specie allows it to survive under limited and harsh conditions, such as low nutrient availability, low oxygen levels, and shear stress forces.

Little is known about the influence of *C. parapsilosis* morphology on antifungal susceptibility. A recent study showed that cellular processes involved in pseudohyphal growth and CAS susceptibility are related when *C. parapsilosis* is grown on glycerol at 37°C but not when grown on glucose (Rupert and Rusche, 2022). Thus, although correlations between pseudohyphal growth and susceptibility to antifungals could exist in *C. parapsilosis*, they may be restricted to particular scenarios. In this study, we evaluated *C. parapsilosis* susceptibility to CAS, the recommended first-line empirical treatment of patients with systemic candidiasis (Tóth et al., 2019), and complemented the results by exploring the polyene AmB. However, under the evaluated conditions, our results do not provide evidence of the mentioned association.

On the other hand, previous studies had described variations in biofilm drug susceptibility according to glucose levels (Santos et al., 2020; Gómez-Molero et al., 2021). For instance, reducing the metabolic activity of *C. parapsilosis* biofilms by 70% requires a concentration of 0.5 µg/mL and 16 µg/mL of AmB when formed on RPMI and YPD medium, respectively (Gómez-Molero et al., 2021). This could be directly related to the fact that increasing glucose concentrations produces *C. parapsilosis* biofilms with higher amounts of carbohydrates in the ECM (Pereira et al., 2015). To test this hypothesis, we broadly estimate the carbohydrate content of biofilms formed on each medium by COMSTAT2 quantification of ConA-A647 stained biomass (**Figure 4**). We noticed that a greater carbohydrate content in both the cell wall and ECM (**Figure 4**) correlates with a lower decrease in biofilm metabolic activity after AmB treatment (**Figures 3B, C**). This is consistent with previous reports of antifungal tolerance correlating with the presence of glucans and mannans on *Candida* biofilm ECM, which decreased the drug’s capacity to penetrate and reach cells (Dominguez et al., 2018; Nett and Andes, 2020). Moreover, we hypothesize that some media could favor an increase in carbohydrate content of the cell wall as rescue mechanism against AmB fungal membrane damage.

Nonetheless, it is important to mention that we used ConA-A647 staining as a first exploratory method for biofilm carbohydrate quantification (a dye specific for α-mannopyranosyl and α-glucopyranosyl residues from polysaccharides), but more accurate techniques must be performed to validate our data. Likewise, it is relevant to assess the influence of culture media on other cell wall and ECM components (chitin, β-1,3 glucan, protein, eDNA, etc.) and evaluate their impact on biofilm susceptibility. By doing so, new combinations of antifungals and specific ECM-disrupting compounds will be identified as a strategic treatment for *C. parapsilosis* biofilm infections.

Finally, we evaluated whether the *in vivo* virulence of *C. parapsilosis* varies according to nutrient availability (**Figure 5**). To that end, we used *G. mellonella*, an alternative animal model suitable for studying *C. parapsilosis* infections (Gago et al., 2014; Jacobsen, 2014; Souza et al., 2015; Binder et al., 2020; Rupert and Rusche, 2022). It was previously reported that the virulence of *C. parapsilosis* cultures on *G. mellonella* is not associated with the metabolic activity and biomass of biofilms formed by the same strains (Marcos-Zambrano et al., 2020). Thus, we injected suspensions from planktonic cultures and biofilms. As far as we know, this is the first report of inoculation of biofilm suspensions into *G. mellonella*. Although we realize this does not represent a chronic infection model, we wanted to determine if metabolic and morphologic adaptations to biofilm state and/or the presence of ECM components exerted variation in virulence depending on the culture media used. Indeed, differences in virulence between planktonic cultures and biofilm suspensions from the same media showed that, depending on nutrient availability, some biofilm factors could favor *C. parapsilosis* pathogenicity (**Figure 4**). For instance, the host immune response: *C. parapsilosis* biofilms are more resistant to neutrophil killing than planktonic cells, probably due to extracellular mannan-glucan (Camarillo-Márquez et al., 2018; Nett and Andes, 2020). Besides, yeast β - (1,3) glucan alters hemocyte subpopulations in *G. mellonella* and enhance their ability to kill (Sheehan and Kavanagh, 2018).

On the other hand, the biofilm state implies metabolic adaptations that can have a role in pathogenicity (Tóth et al., 2019). For example, the hydrolytic lipases are enzymes required for biofilm formation but also host tissue damage and modulation of the immune system (Tóth et al., 2019; Zoppo et al., 2021). Moreover, Rupert & Rusche (2022) found that *G. mellonella* survival was different if *C. parapsilosis* cells were pre-grown on a medium containing glucose or glycerol (Rupert and Rusche, 2022). Thus, *C. parapsilosis* virulence is affected by the carbon source available before infection, suggesting a critical effect of yeast metabolic adaptations on host mortality. Notably, they did not observe any correlation between pseudohyphal growth and virulence outcomes (Rupert and Rusche, 2022).

Filamentation in *C. albicans* is important for tissue invasion; however, a blastospore-locked mutant had shown virulence advantages in a mouse model of systemic infection (Dunker et al., 2021). Similarly, a mutant with hyphal formation defects have shown that filamentation alone is insufficient to kill *G. mellonella* (Fuchs et al., 2010), indicating that blastospores themselves are not less virulent than filamentation forms (Dunker et al., 2021). In fact, most pathogenic dimorphic fungi do not grow as hyphae in the body, like *Histoplasma* spp., *Blastomyces* spp., *Candida auris* and C*andida glabrata* (Sudbery et al., 2004; Galocha et al., 2019; Dunker et al., 2021). In the case of *C. parapsilosis*, pseudohyphae has not been associated with invasion or cell damage (Kim et al., 2006; Silva et al., 2009; Tóth et al., 2018). A hyperfilamentous mutant had shown a downregulation of genes important for pathogenicity and less organ fungal burden on mice (Banerjee et al., 2019). Similarly, a mutated strain with extremely long and aggregating pseudohyphae had increased survival to killing by J774.1 macrophage-like cells but was avirulent on *G. mellonella* and had reduced fungal burden on mice (Tóth et al., 2018).

Our virulence results agree with the previously exposed information (**Figure 5**). Nutrient availability of different culture media influences *G. mellonella* survival after *C. parapsilosis* infection with cells from planktonic cultures or biofilms. As expected, we observed that biofilm suspensions had a more pronounced effect on larvae survival. However, we cannot explain virulence solely by correlating it with one of the studied factors (biomass, metabolic activity, filamentation, carbohydrate content). Instead, we believed that different biological traits contribute to *C. parapsilosis* virulence, with metabolic adaptations being a critical focus for further research. Future work on variations in the expression of specific virulence factors (lipases, proteinases, adhesins, etc.) according to nutrient availability could help narrow the landscape. Notably, TSB media produces biofilms that are more virulent but more susceptible to the evaluated antifungals, suggesting a trade-off in *C. parapsilosis* performance.

Our experiments were performed using a single clinical isolate of *C. parapsilosis*, but we expect the overall findings to be useful and applicable. A detailed outline of culture media influence on *C. parapsilosis* main characteristics are summarized in **Table 1**. This study reaffirms the importance of understanding how pathogenic microorganisms behave under different conditions to establish research protocols that better resemble the infection site. Only in this way we will find appropriate therapeutic strategies. Considering the impact of glucose on *C. parapsilosis* biofilms, it could be recommendable that candidemia studies be performed considering two different scenarios: catheter-associated infections with low glucose concentrations (0.06-0.1% bloodstream) (Dunker et al., 2021) or infections associated with parenteral nutrition with high glucose concentrations (10-30%) (Herek et al., 2019).

## Data availability statement

The raw data supporting the conclusions of this article will be made available by the authors, without undue reservation.

## Author contributions

BA-J: Conceptualization, Data curation, Formal Analysis, Investigation, Methodology, Software, Validation, Visualization, Writing – original draft, Writing – review & editing. JA: Data curation, Formal Analysis, Investigation, Methodology, Writing – review & editing. NB-C: Data curation, Investigation, Methodology, Supervision, Writing – review & editing. ET: Conceptualization, Formal Analysis, Funding acquisition, Investigation, Project administration, Resources, Supervision, Validation, Visualization, Writing – review & editing.
